# Heterodimeric TALENs induce targeted heritable mutations in the crustacean *Daphnia magna*

**DOI:** 10.1242/bio.20149738

**Published:** 2015-02-13

**Authors:** Akiko Naitou, Yasuhiko Kato, Takashi Nakanishi, Tomoaki Matsuura, Hajime Watanabe

**Affiliations:** 1 Department of Biotechnology, Graduate School of Engineering, Osaka University, 2-1 Yamadaoka, Suita, Osaka 565-0871, Japan; 2 Frontier Research Base for Global Young Researchers, Graduate School of Engineering, Osaka University, 2-1 Yamadaoka, Suita, Osaka 565-0871, Japan

**Keywords:** Heterodimeric TALENs, Heritable mutation, Biallelic mutation, *Daphnia magna*, Eyeless, DsRed2

## Abstract

Transcription activator-like effector nucleases (TALENs) are artificial nucleases harboring a customizable DNA-binding domain and a *Fok*I nuclease domain. The high specificity of the DNA-binding domain and the ease of design have enabled researchers to use TALENs for targeted mutagenesis in various organisms. Here, we report the development of TALEN-dependent targeted gene disruption in the crustacean *Daphnia magna*, the emerging model for ecological and toxicological genomics. First, a reporter transgene *DsRed2* (*EF1α-1::DsRed2*) was targeted. Using the Golden Gate method with a GoldyTALEN scaffold, we constructed homodimeric and heterodimeric TALENs containing wild-type and ELD/KKR *Fok*I domains. mRNAs that coded for either the customized homodimeric or heterodimeric TALENs were injected into one-cell-stage embryos. The high mortality of embryos injected with homodimeric TALEN mRNAs prevented us from detecting mutations. In contrast, embryos injected with heterodimeric TALEN mRNAs survived and 78%–87% of the adults lost DsRed2 fluorescence in a large portion of cells throughout the body. In addition, these adults produced non-fluorescent progenies, all of which carried mutations at the *dsRed2* locus. We also tested heterodimeric TALENs targeted for the endogenous *eyeless* gene and found that biallelic mutations could be transmitted through germ line cells at a rate of up to 22%. Both somatic and heritable mutagenesis efficiencies of TALENs were higher than those of the CRISPR/Cas9 system that we recently developed. These results suggest that the TALEN system may efficiently induce heritable mutations into the target genes, which will further contribute to the progress of functional genomics in *D. magna*.

## Introduction

The water flea *Daphnia* is a planktonic crustacean ubiquitously found in fresh water. It occupies an important position in food webs as a link between producer algae and secondary consumers such as small fishes (Miner et al., 2012). *Daphnia* is sensitive to anthropogenic chemicals and environmental changes (Ebert, 2005). For these reasons, *Daphnia* has long been used as a model animal in ecology (Lampert, 2011) and toxicology (Tatarazako and Oda, 2007). In addition, it is an attractive model species for understanding the evolution of reproductive strategies because it usually alternates between parthenogenetic and sexual modes of reproduction with changes in environmental quality (Hebert, 1978). Its significance in ecology, evolutionary biology, and toxicology has prompted us to conduct genetic research on *Daphnia*. Recently, expressed sequence tag (EST) sequences (Watanabe et al., 2005) and a draft genome sequence (Orsini et al., 2011) of *D. magna* were determined. The genome sequence of a related organism, *D. pulex*, has also been described (Colbourne et al., 2011). In addition, targeted gene manipulation using RNA interference (RNAi) by microinjection of double-stranded RNAs (dsRNAs) into eggs has been developed to investigate the relationship between available genetic information and phenotypes in *D. magna* (Kato et al., 2011). However, to overcome the inability of this method to induce null phenotypes and the transient nature of RNAi, it was necessary to develop targeted mutagenesis.

Targetable nucleases are new tools in genome engineering that have been applied in a large range of organisms (Carroll, 2014). Zinc finger nucleases (ZFNs) and transcription activator-like effector nucleases (TALENs) contain a custom-designed DNA-binding domain and a *Fok*I nuclease domain (Pan et al., 2013). Both of the artificial nucleases can generate double strand breaks at the target site that can be repaired by error-prone non-homologous end-joining (NHEJ), resulting in gene disruptions through the introduction of small insertions or deletions (in-dels) (Pan et al., 2013; Carroll, 2014). The other tool, the CRISPR/Cas9 system, can also induce targeted gene disruption by a mechanism distinct from the targeted nucleases described above. In this system, a custom guide RNA (gRNA), a chimeric CRISPR RNA (crRNA) linked to a transactivating crRNA (tracrRNA), directs the endonuclease Cas9 to its cognate target DNA and induces repair of double-strand break, resulting in introduction of indels at the target loci (Jinek et al., 2012). We recently reported that the CRISPR/Cas system could introduce heritable mutations into the endogenous *eyeless* gene in *D. magna* (Nakanishi et al., 2014).

**Fig. 1.**
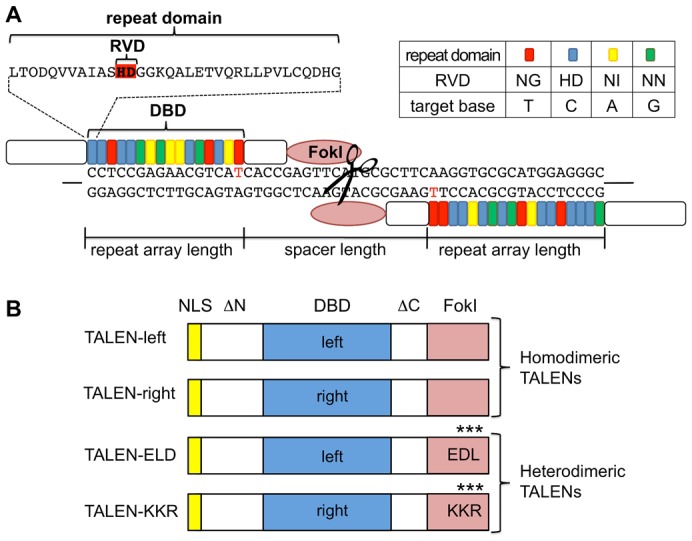
Schematic illustration of the TALEN system. (A) Binding of the customized TALENs on the genome. The DNA-binding domain (DBD) is composed of repeat domains. Each repeat domain consists of 34 a.a. residues and recognizes one nucleotide. Among the 34 a.a. residues, a set of the 12th and 13th residues is referred to as repeat-variable di-residue (RVD) and functions as a determiner for nucleotide recognition of the domain. The repeat domains containing NG, HD, NI, and NN recognize T, C, A, and G and are shown with red, blue, yellow, and green boxes, respectively. Length of the DNA-binding site (named “repeat array length”) and length between two DNA-binding sites (named “spacer length”) were important parameters for target recognition and dimerization of TALENs on the genome. T at position 0 of the DNA-binding sequence was shown in red. (B) Domain structure of homodimeric and heterodimeric TALENs. GoldyTALEN scaffold was used as a backbone. Both TALEN-left and TALEN-right for homodimeric TALENs contain a wild-type *Fok*I domain. TALEN-ELD and TALEN-KKR for heterodimeric TALENs contain a mutated *Fok*I domain ELD and KKR, respectively. ΔN and ΔC indicate truncated N-terminal and C-terminal domains of TALEN. Left and right DBDs were customized to bind in close proximity. All of the TALENs have an SV40 nuclear localization signal (NLS).

Compared with ZFNs harboring a DNA-binding domain that consists of three to four zinc fingers, TALENs have three advantages in targeted mutagenesis: (1) DNA binding specificity is higher, (2) off-target effects are lower, and (3) construction of DNA-binding domains is easier (Pan et al., 2013). The DNA-binding domain of TALENs is derived from TAL effector of plant pathogenic *Xanthomonas* spp. (Bogdanove et al., 2010; Boch and Bonas, 2010) and consists of tandemly repeated domains, each of which contains a highly conserved sequence of 34 amino acids and recognizes a single DNA nucleotide. Among each repeat, amino acids 12 and 13, referred to as repeat-variable di-residue (RVD), determine the target nucleotide of the repeat domain. The RVDs NN, NG, NI, and HD preferentially recognize guanine, thymine, adenine, and cytosine (Boch et al., 2009; Moscou and Bogdanove, 2009). Combinations of the repeats enable TALENs to be bound at specific target sequences. Because of the requirement of *Fok*I domain dimerization for its nuclease activity, co-expression of two TALENs that bind to DNA at a proximal site and to each other is necessary to generate the double-strand breaks at a target site. Recent studies have reported that heterodimerization of the modified *Fok*I domains ELD and KKR reduces TALEN cytotoxicity and increases nuclease activity (Cade et al., 2012; Nakajima and Yaoita, 2013). A schematic illustration of the TALEN system is shown in Fig. 1.

In this study, we aimed to establish TALEN-mediated target mutagenesis in *D. magna*. The GoldyTALEN, a TALEN with truncated N-terminal and C-terminal domains (Bedell et al., 2012), was used as a backbone for the TALENs examined in this study. We selected a hemizygous *DsRed2* reporter gene and an endogenous *eyeless* (*ey*) gene as targets for TALENs. To find an optimal TALEN scaffold that would achieve targeted mutagenesis in *D. magna*, we tested homo- and heterodimeric TALEN scaffolds that contain a homodimer of the wild-type *Fok*I domains and heterodimeric ELD/KKR *Fok*I domains, respectively. We found that homodimeric TALENs showed high toxicity that stopped embryogenesis. In contrast, heterodimeric TALENs were less toxic and introduced in-del mutations at *dsred2* and at *ey* loci in somatic and germ cells. Importantly, both somatic and heritable mutation rates of the TALENs were higher than those of CRISPR/Cas9. Our results indicate that heterodimeric TALENs are useful for inducing targeted mutagenesis in *D. magna*. This method will contribute to further development of functional analyses of the newly discovered genes in this new model species in ecological, evolutionary, and toxicological genomics studies.

## MATERIALS AND METHODS

### *Daphnia* strains

The *Daphnia magna* strain (NIES clone) was obtained from the National Institute for Environmental Studies (NIES, Tsukuba, Japan) and cultured under laboratory conditions for many generations. A transgenic *D. magna* containing a hemizygous *DsRed2* gene under the control of the *D. magna EF1α-1* promoter was generated by microinjection of pCS-*EF1α-1*:*DsRed2* using a previously developed method (Kato et al., 2012). pCS-*EF1α-1*:*DsRed* was constructed by replacing the H2B-GFP coding sequence of pCS-*EF1α-1*::H2B-GFP (Kato et al., 2012) with the *DsRed2* sequence. This transgenic line, which exhibits ubiquitous *DsRed2* expression, has been maintained for more than 50 generations. Characterization of the transgene structure is shown in supplementary material Fig. S1.

### *Daphnia* culture conditions

To minimize variations in maternal effects that could influence microinjection, we maintained the strain under the following conditions: 120 neonates (under 24 h) were transferred to 5 L of ADaM medium (Klüttgen et al., 1994) and cultured at 22–24°C under a light/dark photoperiod of 16 h/8 h. The culture medium was not changed for 3 weeks. Daphniids were fed once per day with 7.5 mg of *Chlorella vulgaris* (Nikkai Center, Tokyo, Japan) and 7.5 mg of baker's yeast (marusanPantry, Ehime, Japan) during the first week; after they matured, their offspring were removed once per day and they were fed 15 mg of *Chlorella* and 15 mg of yeast daily. The addition of yeast helped to maintain the number of juveniles per clutch continuously higher than that observed for those fed *Chlorella* only (data not shown).

### TALEN design

The TALEN target sites were identified using TAL Effector Nucleotide Targeter (TALE-NT) 2.0 (Doyle et al., 2012) with the following parameters: (1) spacer length of 15 to 20 nucleotides, (2) repeat array length of 15 to 20 nucleotides, (3) NN for G substitute, (4) T at position 0, and (5) using guidelines proposed by Streubel et al. (Streubel et al., 2012) (Fig. 1A). The *DsRed2* (*dsr*) TALEN recognition sequences were: left TALEN 5′-GCCCTCCATGCGCACCTT-3′ and right TALEN 5′-CCTCCGAGAACGTCAT-3′. The spacer between the two *dsr*-TALEN binding sites was 19 bp long. The *ey* TALEN recognition sequences are: left TALEN 5′-GTTGTTCTGGTCCACGCC-3′ and right TALEN 5′-GGCGTCGTGAGGAGA-3′. The spacer between the two *ey*-TALEN binding sites was 15 bp long.

### TALEN constructs

Construction of *dsr*-TALEN expression vectors was divided into three steps. First, TALEN assemblies of the RVD-containing repeats were conducted using the Golden Gate approach (Cermak et al., 2011). Once assembled, the RVD-containing repeats were cloned into a pT3TS destination vector with the GoldyTALEN backbone (Addgene plasmid 38142) (Bedell et al., 2012), resulting in construction of pT3TS-*dsr*-TALEN-left and -right. Second, to generate vectors for the synthesis of *dsr*-TALEN mRNAs to be injected into *D. magna* eggs, *dsr*-TALEN-left and -right coding sequences were amplified and cloned between the vasa 5′ UTR and 3′ UTR on pCS-Dmavas-Cas9 for Cas9 mRNA expression in *D. magna* (Nakanishi et al., 2014) by using the In-Fusion PCR cloning kit (Clontech, California, USA). This resulted in construction of pCS-Dmavas-*dsr*-TALEN-left and pCS-Dmavas-*dsr*-TALEN-right. Third, Q486E, I499L, and N496D mutations to *dsr*-TALEN-left and E490K, I538K, and H537K mutations to *dsr*-TALEN-right were introduced by using QuickChange Lightening Multi Site-Directed Mutagenesis Kit (Agilent Technologies, Cedar Creek, TX); this resulted in construction of heterodimeric *dsr*-TALEN expression vectors pCS-Dmavas-*dsr*-TALEN-ELD and –KKR. The mutagenesis was performed using the following oligonucleotides: TALEN-ELD, ELD-1 5′-CAATTGGTCAAGCAGATGAAATGGAAAGATATGTCGAAG-3′ and ELD-2 5′-AGAATCAAACAAGAGACAAGCATCTCAACCCTAATGAATG-3′; and TALEN-KKR, KKR-1 5′-GCAAAGATATGTCAAGGAGAATCAAACAAGAAACAAG-3′ and KKR-2 5′-GCTCAGCTTACAAGATTGAATCGTAAGACTAATTGTAATGGAGC-3′.

Homodimeric *ey*-TALEN expression vectors pT3TS-*ey*-TALEN-left and pT3TS-*ey*-TALEN-right, were constructed and digested with *Xba*I and *Bsa*BI endonucleases. The resulting products encoding RVD-containing repeats were cloned into the *Xba*I/*Bsa*BI site of pCS-Dmavas-*dsr*-TALEN-ELD and pCS-Dmavas-*dsr*-TALEN-KKR, resulting in construction of heterodimeric *ey*-TALEN expression vectors pCS-Dmavas-*ey*-TALEN-ELD and -KKR, respectively.

### *In vitro* transcription

All of the TALEN expression vectors were linearized with *Acc*65I endonuclease, purified by phenol-chloroform extraction, and used as templates for *in vitro* transcription. To synthesize capped RNA, each template was subjected to *in vitro* transcription with the mMessage mMachine® SP6 kit (Life Technologies, California, USA). Poly (A) tails were attached to each mRNA using a Poly(A) Tailing Kit (Life Technologies), according to the manufacturer's instructions. *In vitro*-synthesized RNAs were column purified using mini Quick Spin RNA columns (Roche Diagnostics GmbH, Mannheim, Germany), followed by phenol/chloroform extraction, ethanol precipitation, and dissolution in DNase/RNase-free water (Life Technologies).

### Microinjection

*In vitro* synthesized RNAs were injected into *Daphnia* eggs according to established procedures (Kato et al., 2011). Briefly, the eggs were collected just after ovulation from 2- to 3-week-old daphniids and placed in ice-chilled M4 medium containing 80 mM sucrose (M4-sucrose). The synthesized RNAs, which contained the water-soluble fluorescent dye Lucifer Yellow (1 mM; LY, Life Technologies) were prepared. LY was used to quantify the injection volume 1 h after microinjection and to distinguish eggs injected with RNA from the unmodified eggs (Törner et al., 2014). The synthesized RNAs were injected through a glass needle under constant pressure of nitrogen gas. Finally, an injected egg was transferred into each well of a 96-well plate filled with 100 µl of M4-sucrose. Microinjections were carried out within an hour after ovulation.

### Photography of daphniids

*DsRed2* expression was detected using a Leica M165C fluorescence stereoscopic microscope (Leica Microsystems Heidelberg GmbH, Mannheim, Germany) equipped with a 545-nm excitation and a 620-nm barrier filter (DSR filter set). Light field and fluorescent images were recorded with a color digital camera (Leica DC500; Leica Microsystems) mounted on the microscope.

## Results

### Design of homo- and heterodimeric *dsr*-TALENs for the reporter gene *DsRed2* in transgenic *D. magna*

First, we targeted a hemizygous reporter gene *EF1α-1::DsRed2* that was mapped to the scaffold03169 (supplementary material Fig. S1) because loss of the DsRed2 fluorescence would allow us to find cell populations that contained the mutated *DsRed2* gene. A homodimeric TALEN pair for *DsRed2* (*dsr*-TALEN-left and –right) was constructed based on a GoldyTALEN scaffold (Bedell et al., 2012) using the Golden Gate assembly method (Cermak et al., 2011). DNA binding domains of homodimeric *dsr*-TALEN-left and –right were customized to bind the 5′-side of the *DsRed2* open reading frame (ORF) with spacing distance of 19 bp (Fig. 1A; supplementary material Fig. S1). In addition, we generated a heterodimeric *dsr*-TALEN pair (*dsr*-TALEN-ELD and –KKR) by introducing ELD/KKR mutations into *Fok*I domains of the homodimeric *dsr*-TALEN pair. All of the customized *dsr*-TALEN coding sequences were linked to UTRs of *D. magna vasa* (Dmavas) exclusively expressed in germ cells (Sagawa et al., 2005) and the resulting chimeric RNAs were subsequently synthesized *in vitro*. Structural differences between homodimeric and heterodimeric TALENs are shown in Fig. 1B.

### Toxicity of homodimeric *dsr*-TALENs to *D. magna* embryos

**Fig. 2.**
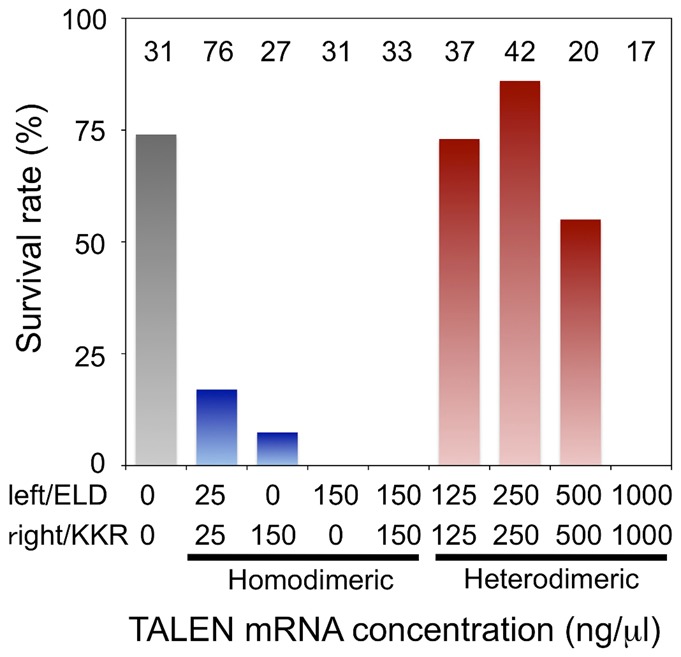
Survival rate of daphniids injected with *dsr*-TALEN mRNAs. TALEN-left and TALEN-right mRNAs for homodimeric TALENs or TALEN-ELD and TALEN-KKR mRNAs for heterodimeric TALENs were injected into eggs just after ovulation and the survival rates of the injected daphniids were investigated at the first-instar juvenile stage. The number of injected daphniids is indicated at the top of each column.

Homodimeric *dsr*-TALEN pair mRNAs were co-injected into one-cell embryos with 1 mM Lucifer Yellow that was used to confirm if the injection volume was constant during the experiment. After culturing for 3 d, the survival rate of mRNA-injected embryos was calculated at the first-instar juvenile stage (Fig. 2; Table 1). An amount of 150 ng/µl of each of the homodimeric *dsr*-TALEN pair mRNAs was lethal in all of the injected embryos. A separate injection of 150 ng/µl of homodimeric *dsr*-TALEN-left and -right mRNA also stopped development during embryogenesis (Fig. 2; Table 1). Even with the injection of 25 ng/µl of each of the mRNAs, survival rate was 17%. The surviving daphniids neither showed phenotypic changes nor produced non-fluorescent G_1_ daphniids (Table 1). These results suggested that the homodimer of a wild-type *Fok*I domain conferred severe toxicity to *D. magna* embryos.

**Table 1.**
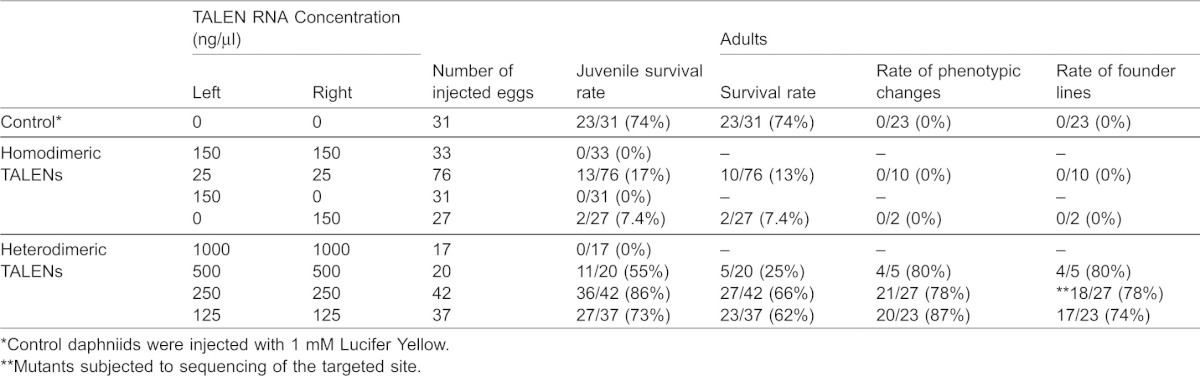
Summary of phenotypes induced by homo- and heterodimeric *dsr2*-TALENs

### Heritable mutagenesis of the transgene *DsRed2* by heterodimeric TALENs

Heterodimeric *dsr*-TALEN pair mRNAs were injected and the survival rate of injected embryos was calculated at the first-instar juvenile stage. In contrast to homodimeric *dsr*-TALENs, 125 and 250 ng/µl of each heterodimeric *dsr*-TALEN pair mRNAs allowed the injected embryos to develop at rates of 73% and 86%, respectively; these rates were similar to the survival rates caused by microinjection of LY without RNA. A concentration of 500 ng/µl decreased the survival rate to 55% and 1000 ng/µl was the absolute lethal concentration.

**Fig. 3.**
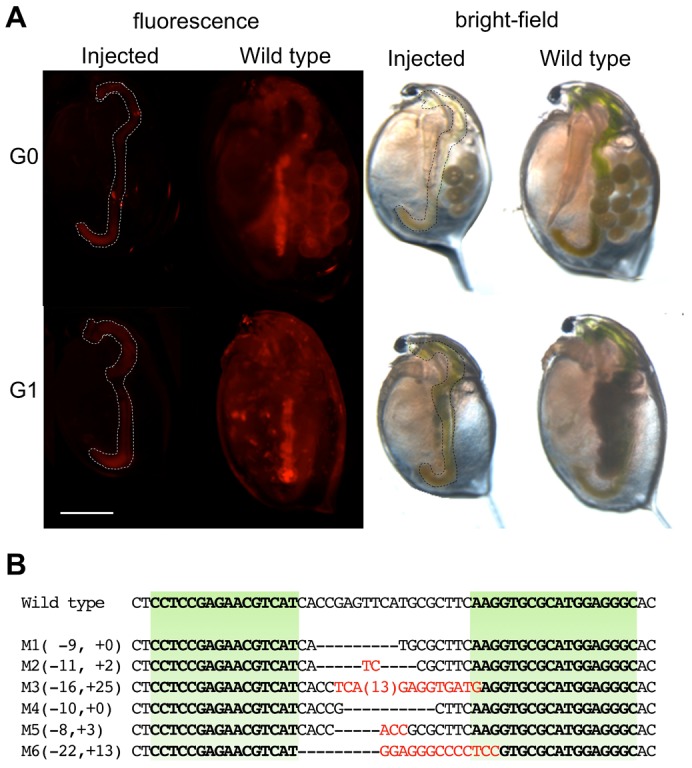
Knockout of a hemizygous *DsRed2* gene. (A) *DsRed2* expression. Upper and lower columns show G_0_ and G_1_ adults. Images were taken from the same sample under a fluorescence microscope (left image) and a bright-field microscope (right image). Each panel contains the TALEN-left and -right mRNAs injected animals (Injected) and the wild-type daphniids (Wild type). The regions surrounded by dashed lines are digestive tracts with observed autofluorescence of *Chlorella* that was used as a food for daphniids. Scale bar: 2 mm. (B) Genome sequences of *DsRed2* knock out mutant lines around the TALEN-targeted site. In the alignment, the top line represents the wild-type *DsRed2* sequence, and subsequent lines show sequences from six different G_1_ mutants (see Table 1). The target sites for TALENs are indicated in green. The length (in bp) of each indel mutation is written to the left of each sequence (−, deletions; +, insertions). In each mutant sequence, deletions are indicated by gaps, insertions by red letters, sequences corresponding to the wild-type targeted sequence are in bold, and the length (in bp) of an abbreviated sequence is given within parentheses.

At the first-instar juvenile stage, no decrease in DsRed2 fluorescence was detected in any of the surviving juveniles, possibly because of the stability of the DsRed2 protein that accumulated maternally and during early embryogenesis. This led to further observations at later stages of development. After the injected animals matured, approximately 80% of daphniids injected with heterodimeric TALEN pair-mRNAs lost their DsRed2 fluorescence in most cells throughout the body at all tested mRNA concentrations (Table 1), suggesting the strong ability of heterodimeric TALENs to introduce somatic mutations (Fig. 3A).

We also found the founder G_0_ animals that produced non-fluorescent G_1_ daphniids were generated by the heterodimeric TALENs, suggesting that the mutations of the *Dsred2* gene were heritable. To calculate the efficiency of inducing heritable mutations, we counted the number of founder G_0_ animals (Fig. 3A). The ratio of founder G_0_ animals to surviving adults was 67%–80%. These results suggested that the heterodimeric TALENs functioned efficiently not only in somatic cells but also in germ line cells.

To investigate how in-del mutations were introduced into the *dsRed2* loci, we collected non-fluorescent G_1_ daphniids from six founder G_0_ animals and extracted their genomic DNAs (Table 1). Genomic PCR products encompassing the TALEN-targeted sites were cloned and sequenced. All of the non-fluorescent G_1_ daphniids had in-del mutations (Fig. 3B). These knockout lines have been maintained for more than 10 generations. Thus, we concluded that the TALENs could induce heritable mutations in the *dsRed2* locus of the transgenic *D. magna*.

### Heritable mutagenesis of the endogenous gene *eyeless* by heterodimeric TALENs

We further examined whether heterodimeric TALENs can introduce mutations in the endogenous gene *eyeless* (*ey*). We previously knocked down and knocked out the *ey* gene using RNAi and CRISPR/Cas-mediated gene disruption (Nakanishi et al., 2014) and found that *D. magna ey* (*Dma-ey*) controls eye development. Heterodimeric *ey*-TALENs were customized to bind to the homeobox region of the *ey* ORF at spacing distances of 15 bp, which partially overlapped with a region previously targeted by the CRISPR/Cas9 system (supplementary material Fig. S2).

**Fig. 4.**
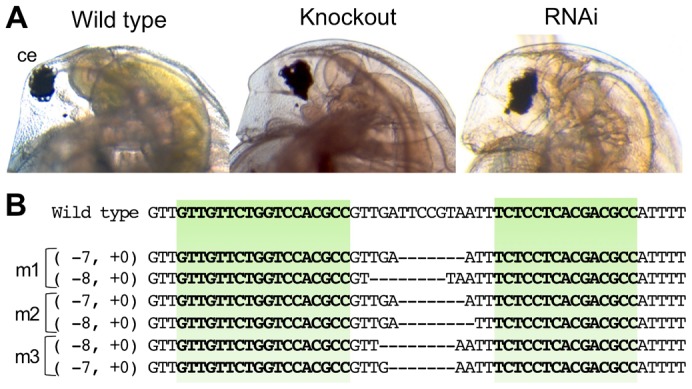
Knockout of an endogenous gene *ey*. (A) Typical phenotypes of *Dma-ey*-deficient daphniids. The images to the left, right, and in the center show the lateral head parts of the wild-type daphniid, *ey* knocked-out daphniid by the TALENs, and *ey* RNAi daphniid generated in a previous study, respectively. Ventral side is left. The abbreviation “ce” refers to compound eye. (B) Genome sequences of *ey*-knock out mutants around TALEN-targeted sites. In the alignment, the top line in the lower alignment represents the wild-type *ey* sequence, and subsequent lines show sequences of three mutant alleles (see Table 2). The target sites for TALENs are indicated in green. The length (in bp) of each indel mutation is written to the left of each sequence (−, deletions; +, insertion). In each mutant sequence, deletions are indicated by underbars, and sequences corresponding to the wild-type targeted sequences are in bold.

Concentrations 125 and 250 ng/µl of each of the heterodimeric *ey*-TALEN-left and -right mRNAs allowed the injected embryos to develop at a rate of 41% and 48%, respectively, and 53% and 78% of the surviving juveniles, respectively, and showed abnormal eye morphology (Table 2). This phenotype was equal to the deformed-eye phenotypes induced by *ey* RNAi and by CRISPR/Cas-mediated *ey* knockout in a previous study (Fig. 4A), suggesting that the heterodimeric *ey*-TALENs functioned in *D. magna*.

**Table 2.**
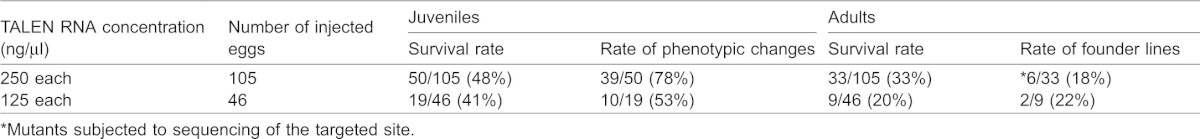
Summary of phenotypes induced by heterodimeric *ey*-TALENs

The founder G_0_ animals that produced deformed-eye G_1_ progenies were generated at a rate of 18%–22% of the surviving adults (Table 2; Fig. 4A). All of the founder G_0_ animals produced non-viable progenies that died within a week. The lethality of *ey* knockout mutants was consistent with that of mutants generated by the CRISPR/Cas9 system (Nakanishi et al., 2014). We sequenced the TALEN-targeted site on the genomes of G_1_ progenies from three founder G_0_ animals. All of the G_1_ progenies had biallelic in-del mutations, resulting in frameshift mutations in both alleles (Fig. 4B). In contrast, monoallelic in-del mutations were found in normal eye G_1_ progenies (data not shown). Thus, we concluded that the heterodimeric TALENs could induce heritable mutations at the endogenous *ey* locus.

## Discussion

Here, we report heritable targeted mutagenesis by heterodimeric TALENs in the cladoceran crustacean *Daphnia magna*. Although all of the eyeless mutant progenies were lethal due to the necessity of its function during development, *dsred2*-knockout lines could be obtained by microinjection of heterodimeric TALEN pair mRNAs and could be maintained for more than 10 generations. This is a simple and efficient method for targeted gene disruption and allows generation of homozygous null mutants without any crossing, and these mutants can be maintained unchanged for generations. These features would be beneficial in studies of parthenogenetic animals, including *Daphnia*, as discussed in our previous study (Nakanishi et al., 2014). In contrast, homodimeric TALENs induced higher mortality during embryogenesis, which prevented us from detecting mutations. Because separate injection of homodimeric TALEN-left and -right mRNAs also stopped embryogenesis, undesired pairing between two identical TALENs via a wild-type *Fok*I domain might have caused off-target cleavage events, as suggested in previous studies (Cade et al., 2012; Nakajima and Yaoita, 2013; Doyon et al., 2011). We conclude that in *D. magna*, heterodimeric TALENs are more suitable for targeted mutagenesis than the homodimeric TALENs.

Heterodimeric TALENs were targeted to the homeobox region of the *ey* gene as was the CRISPR/Cas9-mediated *ey* gene disruption we performed previously (Nakanishi et al., 2014), allowing us to compare the efficiency of the TALENs and the CRISPR/Cas9 system to introduce mutations (Table 3). There was no apparent difference between the two systems in timing of onset of the effects of mutagenesis, because TALENs worked during embryogenesis and led to production of juveniles with abnormal eye morphology as did the CRISPR/Cas9 system. The somatic and heritable mutagenesis efficiencies of TALENs were higher than those of the CRISPR/Cas9 system, although optimal concentrations of gRNAs and Cas9 mRNAs need to be further examined. Therefore, for target mutagenesis of a single gene, TALENs might be more reliable and efficient in *D. magna*. However, for multiple-target mutagenesis, the CRISPR/Cas9 system would be more suitable because of its simplicity of gRNA design.

**Table 3.**
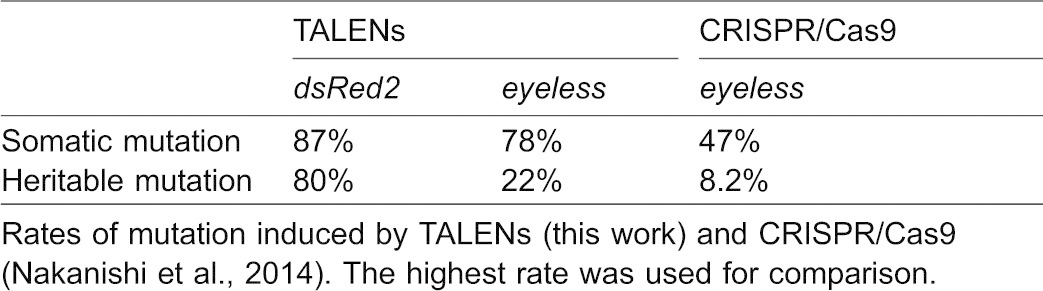
Efficiency of TALENs and CRISPR/Cas9 system for inducing target mutagenesis

Heterodimeric TALENs could introduce somatic mutations in both *ey* and *DsRed2* genes at a ratio of more than 80%. However, the heritable mutation rate differed between the genes (78% in *DsRed2* and 22% in *ey*). In contrast to the high expression of *DsRed2* regulated by the *D. magna EF1α-1* promoter, no or low *eyeless* expression in germ cells was assumed. The efficiency of TALEN-mediated double-strand breaks might be lower in the silenced chromatin than in active chromatin, which might lead to differences in the TALEN activity in germ cells.

Our findings that heterodimeric TALENs work efficiently in *D. magna* will contribute to further development of a TALEN-based genome editing technology for integration of foreign DNAs, as reported in other organisms (Carroll, 2014). *Daphnia* genome engineering will help facilitate use of the emerging daphniid EST and genomic sequences. In turn, this will lead to a better understanding of the ecology, evolutionary biology, and molecular biology of *Daphnia*.

## Supplementary Material

Supplementary Material
