# Supplementary material for: Heterodimeric TALENs induce targeted heritable mutations in the crustacean Daphnia magna
Source: Biol Open. 2015 Feb 13;4(3):364–9. doi: 10.1242/bio.20149738 (PMC4359742; doi:10.1242/bio.20149738)
Supplement: Supplementary Material [file supp_4_3_364__index.html]

Heterodimeric TALENs induce targeted heritable mutations in the crustacean Daphnia magna — Heterodimeric TALENs induce targeted heritable mutations in the crustacean Daphnia magna — Supplementary Material 

# Heterodimeric TALENs induce targeted heritable mutations in the crustacean *Daphnia magna*

## bio.20149738 Supplementary Material

**Files in this Data Supplement:**

- Supplementary Material - Akiko Naitou et al. doi: 10.1242/bio.20149738
