# Supplementary figures and images for: Heterodimeric TALENs induce targeted heritable mutations in the crustacean Daphnia magna
Source: Biol Open. 2015 Feb 13;4(3):364–9. doi: 10.1242/bio.20149738 (PMC4359742; doi:10.1242/bio.20149738)

Supplementary Material  
Akiko Naitou et al. doi: 10.1242/bio.20149738

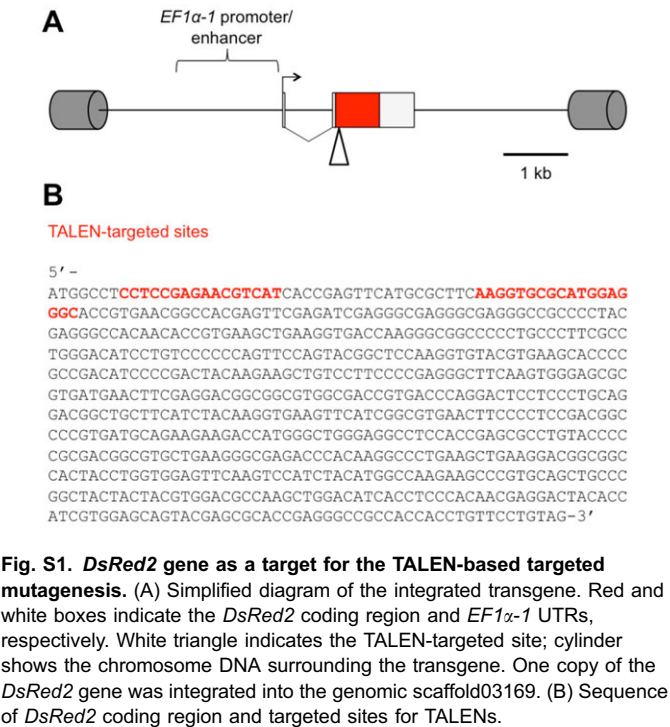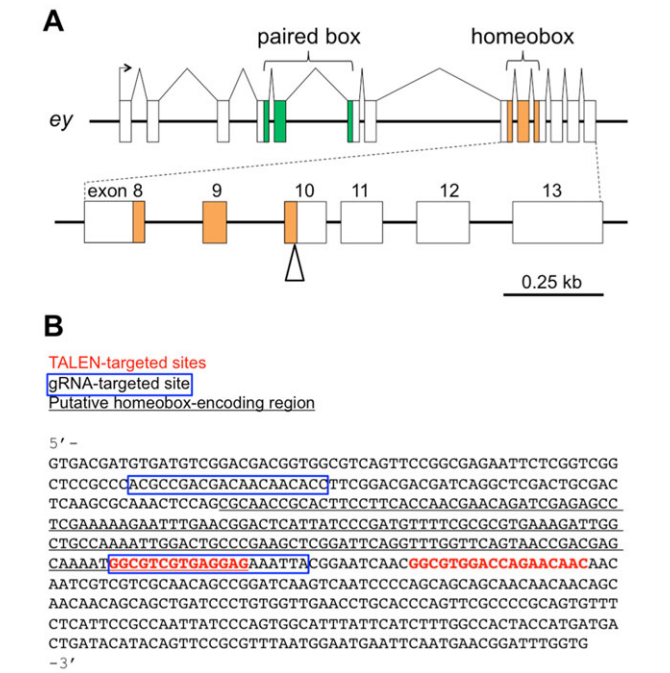

Supplement: Supplementary Material [file supp_bio.20149738_bio.20149738-s1.pdf]
